# Long Non-Coding RNA Malat-1 Is Dispensable during Pressure Overload-Induced Cardiac Remodeling and Failure in Mice

**DOI:** 10.1371/journal.pone.0150236

**Published:** 2016-02-26

**Authors:** Tim Peters, Steffie Hermans-Beijnsberger, Abdelaziz Beqqali, Nicole Bitsch, Shinichi Nakagawa, Kannanganattu V. Prasanth, Leon J. de Windt, Ralph J. van Oort, Stephane Heymans, Blanche Schroen

**Affiliations:** 1 Center for Heart Failure Research, Department of Cardiology, CARIM School for Cardiovascular Diseases, Maastricht University, Maastricht, The Netherlands; 2 Department of Experimental Cardiology, Academic Medical Center, Amsterdam, The Netherlands; 3 Department of Cardiology, CARIM School for Cardiovascular Diseases, Maastricht University, Maastricht, The Netherlands; 4 RNA Biology Laboratory, RIKEN, Wako, Saitama, Japan; 5 Department of Cell and Developmental Biology, University of Illinois at Urbana-Champaign, Urbana, IL, United States of America; 6 Netherlands Heart Institute (ICIN), Utrecht, The Netherlands; 7 Centre for Molecular and Vascular Biology (CMVB), Department of Cardiovascular Sciences, Katholieke Universiteit Leuven, Leuven, Belgium; Albert Einstein College of Medicine, UNITED STATES

## Abstract

**Background:**

Long non-coding RNAs (lncRNAs) are a class of RNA molecules with diverse regulatory functions during embryonic development, normal life, and disease in higher organisms. However, research on the role of lncRNAs in cardiovascular diseases and in particular heart failure is still in its infancy. The exceptionally well conserved nuclear lncRNA Metastasis associated in lung adenocarcinoma transcript 1 (Malat-1) is a regulator of mRNA splicing and highly expressed in the heart. Malat-1 modulates hypoxia-induced vessel growth, activates ERK/MAPK signaling, and scavenges the anti-hypertrophic microRNA-133. We therefore hypothesized that Malat-1 may act as regulator of cardiac hypertrophy and failure during cardiac pressure overload induced by thoracic aortic constriction (TAC) in mice.

**Results:**

Absence of Malat-1 did not affect cardiac hypertrophy upon pressure overload: Heart weight to tibia length ratio significantly increased in WT mice (sham: 5.78±0.55, TAC 9.79±1.82 g/mm; p<0.001) but to a similar extend also in Malat-1 knockout (KO) mice (sham: 6.21±1.12, TAC 8.91±1.74 g/mm; p<0.01) with no significant difference between genotypes. As expected, TAC significantly reduced left ventricular fractional shortening in WT (sham: 38.81±6.53%, TAC: 23.14±11.99%; p<0.01) but to a comparable degree also in KO mice (sham: 37.01±4.19%, TAC: 25.98±9.75%; p<0.05). Histological hallmarks of myocardial remodeling, such as cardiomyocyte hypertrophy, increased interstitial fibrosis, reduced capillary density, and immune cell infiltration, did not differ significantly between WT and KO mice after TAC. In line, the absence of Malat-1 did not significantly affect angiotensin II-induced cardiac hypertrophy, dysfunction, and overall remodeling. Above that, pressure overload by TAC significantly induced mRNA levels of the hypertrophy marker genes *Nppa*, *Nppb* and *Acta1*, to a similar extend in both genotypes. Alternative splicing of Ndrg2 after TAC was apparent in WT (isoform ratio; sham: 2.97±0.26, TAC 1.57±0.40; p<0.0001) and KO mice (sham: 3.64±0.37; TAC: 2.24±0.76; p<0.0001) and interestingly differed between genotypes both at baseline and after pressure overload (p<0.05 each).

**Conclusion:**

These findings confirm a role for the lncRNA Malat-1 in mRNA splicing. However, no critical role for Malat-1 was found in pressure overload-induced heart failure in mice, despite its reported role in vascularization, ERK/MAPK signaling, and regulation of miR-133.

## Introduction

The complexity of an organism is not related to the size of its genome nor to the number of proteins encoded therein but rather correlates with the number of genes that produce non-coding RNA [1, 2]. Long non-coding RNAs (LncRNAs) were discovered in the early 1990's [3, 4] and are nowadays defined as RNA molecules of >200 nucleotides in length, lacking a significant open reading frame. They are able to bind other RNA or DNA species as well as proteins and may thereby regulate processes at all stages from gene transcription and translation to protein function. The functions of many lncRNAs in transcriptional regulation have recently attracted much attention in the field of developmental biology (reviewed in [5]) and cancer research (reviewed in [6]). However, also cardiovascular research is beginning to recognize the importance of lncRNAs for heart development (e.g. SRA1) and pathology (e.g. MIAT) as recently reviewed [7, 8]. The lncRNA Metastasis-associated adenocarcinoma transcript 1 (Malat-1; also known as Nuclear Enriched Abundant Transcript 2, Neat2) was discovered in metastasizing non-small cell lung cancer [9] and is highly expressed in most cell types and organs, including the heart. Increased expression of Malat-1 is by now recognized as an established feature of many tumors and indicates poor prognosis [10, 11]. Malat-1 can bind to active chromatin sites [12] and co-localizes with nuclear speckles, where it regulates pre-mRNA splicing [13, 14]. Above that, a cis-regulatory role has been assigned to the Malat-1 locus [15]. Given the high conservation and expression level of Malat-1, several research groups simultaneously undertook the effort to generate Malat-1 knockout mice. Surprisingly but consistently, none of the strains lacking Malat-1 showed any obvious abnormalities during embryonic or post-natal development [15–17], indicating that Malat-1 is either completely dispensable or becomes important only under pathological conditions.

Heart failure (HF) is a condition in which the heart is unable to sustain sufficient blood flow through the body and is the fatal end stage of many heart diseases. About 23 million people worldwide suffer from HF, with the highest prevalence in the growing elderly population [18]. A common cause for HF is cardiac pressure overload, which can be a consequence of hypertension or aortic valve stenosis. Pressure overload of the left ventricle (LV) causes a transient increase in vascularization that is necessary for adaptive cardiomyocyte growth and myocardial vessel density correlates with cardiac function [19–21]. Importantly, genetic ablation of Malat-1 has recently been shown to reduce revascularization capacity after hind limb ischemia [22]. Above that, two groups have shown impaired myogenic differentiation after silencing of Malat-1 *in vitro* [23, 24], possibly via regulation of microRNA-133. This microRNA has central roles in cardiac contractility and hypertrophy by repressing β_1_-adrenergic receptor and serum response factor (SRF), respectively [25, 26]. Scavenging of miR-133 by Malat-1 may therefore increase levels of SRF, an important mediator of cardiac hypertrophy [27]. Similarly, ERK/MAPK signaling propagates pro-hypertrophic signaling in the heart [28] and Malat-1 was found to activate this pathway [29]. These reports strongly suggest a role for Malat-1 in the development of cardiac hypertrophy and failure. Therefore, we subjected Malat-1 knockout mice to either thoracic aortic constriction (TAC) or chronic infusion of angiotensin II (AngII) to induce pressure overload of the LV, mimicking aortic valve stenosis or systemic hypertension, respectively. Surprisingly, detailed analysis of cardiac morphology, function, and histology did not reveal an implication of Malat-1 in myocardial hypertrophy, angiogenesis, inflammation, fibrosis, or dysfunction upon chronic cardiac pressure overload.

## Methods

### Mouse models

Heterozygous Malat-1^+/-^ mice derived from CBA x C57Bl/6 chimeric animals were provided by Shinichi Nakagawa after 6 backcrosses into C57Bl/6N mice [17]. The offspring was genotyped before the start of the studies to match group sizes and only homozygous Malat-1^+/+^ and Malat-1^-/-^ mice were used. DNA was isolated from toes of new born mice and genotyping PCR was performed using a standard 3-step protocol with 30 cycles and 62–66°C annealing temperature. Primer sequences were: WT-Fw AGAGCAGAGCAGCGTAGAGC, WT-Rev GCTCTGGTCAGCCTCCATTA, KO-Fw TTGAAGTGGCGAGCGATAC, and KO-Rev AGATCCCAGCGGTCAAAAC.

Mice were operated at 8–12 weeks of age to induce cardiac hypertrophy by either continuous infusion of angiotensin II (AngII, 2.5 mg∙kg^-1^∙d^-1^) using osmotic minipumps (Alzet) or thoracic aortic constriction (TAC) between the brachiocephalic artery and the left common carotid artery. Aortic diameter was reduced to 0.41 mm (27G needle) for mice weighing up to 25.5 g or to 0.46 mm (26G needle) for larger animals. TAC mimics the situation in patients with aortic valve stenosis with pure pressure overload but without direct hormonal effects. On the other hand, AngII infusion causes systemic hypertension and additionally has direct cellular effects in heart tissue which further promote heart failure [30]. In both studies the duration of the experiment was 4 weeks and sham operated animals served as controls. Conscious heart rate and blood pressure of mice enrolled in the AngII study were measured by CODA tail cuff (Kent Scientific) three weeks after surgery. After four weeks of pressure overload, echocardiography was performed on a Vevo2100 system (Visualsonics) to acquire M-mode images at the height of the papillary muscles. Afterwards, animals were sacrificed to harvest organs for histological analysis and RNA isolation. All animal experiments were carried out in accordance with Dutch law and approved by the animal experimental committee at Maastricht University (permit number 2012–007).

### Histology

After sacrifice, organs were rinsed in PBS and Zinc fixed for 48 hours (BD Pharmingen, #552658). 4 μm paraffin sections were cut to analyze histological changes of the left ventricle and septum. Collagen was stained with Picrosirius Red F3B (Klinipath, #80115) and interstitial collagen area was quantified after exclusion of vessels and endo- and epicardial connective tissue. Laminin staining was performed using rabbit anti-mouse laminin (Sigma, L9393, 1:100) and Vectastain Elite ABC kit (Vector laboratories) to assess cardiomyocyte size. Epicardial cardiomyocytes as well as longitudinal cells and cells without visible nucleus were excluded from cell size analysis. CD45 positive cells were stained with rat anti-mouse CD45 antibody (BD Pharmingen, #553076, 1:500) and Vectastain ABC-AP kit (Vector laboratories) and counted in the whole LV and septum. Capillaries were stained with biotinylated Griffonia (Bandeiraea) Simplicifolia Lectin I (Vector Laboratories, B-1105, 20 μg/mL) and Vectastain ABC-AP kit and cross-sectioned capillaries near the endocardium were counted. All analysis was performed in a blinded manner using a Leica DM2000 equipped with a Leica DFC450C camera and ImageJ software.

### RT-PCR and analysis of alternative splicing

Myocardial RNA was isolated using mirVana microRNA isolation kit (Ambion) according to manufacturer’s instructions. RNA was reverse transcribed using iScript RT kit (Biorad) and RT-qPCR was performed using SYBR Green (Biorad). Primer sequences for Malat-1 and for cardiac hypertrophy markers Atrial natriuretic peptide (*Nppa*), Brain natriuretic peptide (*Nppb*) and Skeletal alpha actin (*Acta1*) were: *Malat-1*_Fw CTTTTCCCCCACATTTCCAA, *Malat-1*_Rev CTCGTGGCTCAAGTGAGGTG; *Nppa*_Fw ATTGACAGGATTGGAGCCCAGAGT, *Nppa*_Rev TGACACACCACAAGGGCTTAGGAT; Nppb_Fw GTTTGGGCTGTAACGCACTGA, *Nppb*_Rev GAAAGAGACCCAGGCAGAGTCA; *Acta1*_Fw TGAGACCACCTACAACAGCA, *Acta1*_Rev CCAGAGCTGTGATCTCCTTC. Gene expression was normalized to Cyclophilin-A (*Ppia*) as internal control: *Ppia*_Fw CAAATGCTGGACCAAACACAA, *Ppia*_Rev GCCATCCAGCCATTCAGTCT.

Alternative mRNA splicing of N-myc downstream-regulated gene 2 (*Ndrg2*) and Eukaryotic translation initiation factor 4H (*Eif4h*) has been reported in hypertrophic and failing mouse hearts, respectively [31, 32]. 500 ng of RNA was reverse-transcribed using Oligo(dT) primers and Superscript II reverse transcriptase (Invitrogen). Primers based on mouse *Ndrg2* (exon 1 Fw: TCAAAGGCAAGTGAAGGTGG, exon 4 Rev: CGAGCCATAAGGTGTCTCCA) and *Eif4h* (exon 3 Fw: GTGGATTCCCTGAAGGAGGC, exon 6 Rev: GAAAGCGACTCCCCATTGGA) were used to detect splicing changes. PCR amplification was performed at 58°C for 30 and 35 cycles, respectively. Electrophoretically separated PCR products were quantified by densitometric analysis using ImageJ software and the ratio of the mRNA isoforms (long/short) was calculated.

### Statistics

Data are presented as median and range with individual data points depicted. Statistical analysis was performed using Prism (GraphPad) and SPSS (IBM). Equality of variances was tested by Levene’s test. One-way ANOVA (with Welch’s correction if appropriate) followed by Tukey or Games-Howell post-hoc test was deployed to compare groups with equal or unequal variances, respectively. In all cases a p-value <0.05 was considered statistically significant.

## Results

### Pressure overload-induced heart failure develops independently of Malat-1

**Fig 1:**
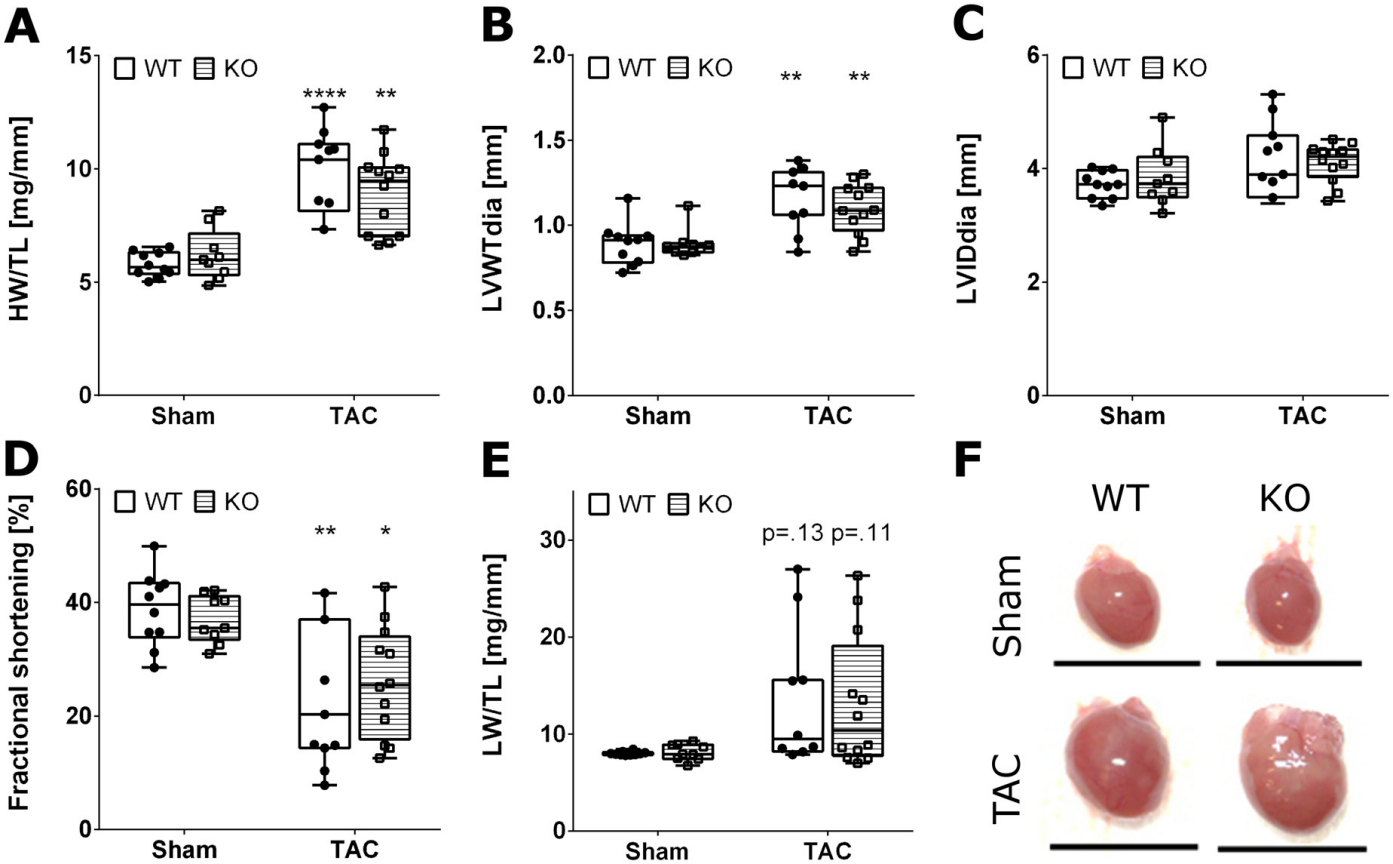
Gross morphological and functional analysis of Malat-1 WT and KO hearts after TAC. Increased heart weight/tibia length (A) and diastolic LV wall thickness (B) confirm concentric hypertrophy in both Malat-1 WT and KO mice without effects on diastolic LV inner diameter (C). Decreased fractional shortening (D) and lung congestion with increased lung weight/tibia length in some animals (E) indicate heart failure independent of Malat-1 deficiency. (F) Representative photographs of mouse hearts, Scale bar: 1 cm; *p<0.05, **p<0.01, ****p<0.0001 TAC versus Sham.

Four weeks after TAC surgery, the effect of LV pressure overload on cardiac dimensions and function were assessed by echocardiography. Both Malat-1 WT and KO mice showed a significant increase in cardiac mass and wall thickness (Fig 1A–1C). Impaired heart function was evidenced by reduced fractional shortening of the left ventricle and in some cases by backward failure leading to lung edema (Fig 1D and 1E). Importantly, the degree of hypertrophy and dysfunction did not significantly differ between WT and Malat-1 KO mice. Similarly, chronic infusion of angiotensin II for four weeks induced comparable degrees of hypertrophy and left ventricular dysfunction in Malat-1 WT and KO mice (S1A–S1F Fig). A summary of animal characteristics, including organ weights can be found in S1 Table. In conclusion, absence of Malat-1 in mice does not affect the development of heart failure upon pressure overload.

### Myocardial remodeling during pressure overload is independent of Malat-1

**Fig 2:**
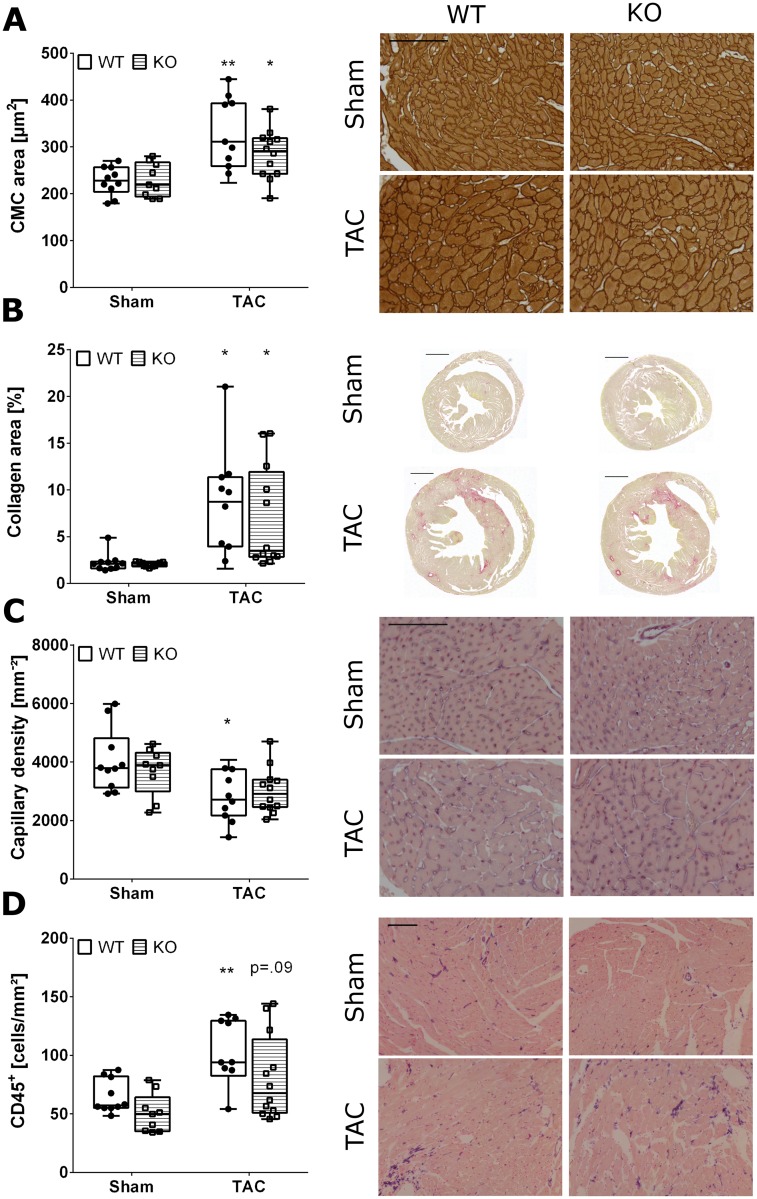
Histological analysis of LV and septal myocardium after TAC. Significant cardiomyocyte hypertrophy (A) and interstitial fibrosis (B) were induced by TAC, whereas the density of endocardial capillaries was mildly decreased (C). (D) Infiltration of CD45 positive leukocytes was apparent after TAC, although this did not reach statistical significance in Malat-1 KO mice. Importantly, no significant differences could be detected between Malat-1 WT and KO mice for any of the histological parameters. *p<0.05, **p<0.01 TAC versus Sham. Scale bars: Sirius Red: 1 mm; all other stains: 100 μm.

Hallmarks of maladaptive cardiac remodeling, including cardiomyocyte hypertrophy, interstitial fibrosis, capillary density, and immune cell infiltration was assessed in heart tissue slides four weeks after induction of pressure overload. Hypertrophy of cardiomyocytes was apparent upon TAC and was unaffected by knockout of Malat-1 (Fig 2A). Similarly, AngII caused a comparable increase in cardiomyocyte area in both Malat-1 WT and KO mice (S2A Fig). The fraction of interstitial collagen area as assessed by staining with Sirius Red was increased significantly after both TAC and AngII, and was independent of Malat-1 expression (Fig 2B and S2B Fig). Capillary density was significantly reduced by TAC in WT mice and showed a similar trend in Malat-1 KO mice, signifying the transition towards heart failure (Fig 2C). However, no significant difference could be found between the two genotypes, suggesting no effect of Malat-1 deficiency on myocardial microvascular perfusion during pressure overload. Finally, while TAC increased the influx of CD45^+^ immune cells into the heart, Malat-1 deficiency did not affect the number of cardiac CD45^+^ cells (Fig 2D). In this genetic background, AngII had milder effects on capillary density and immune cell infiltration than TAC, but again no effects of Malat-1 were identified (S2C and S2D Fig). Taken together, these findings show that Malat-1 is dispensable for cardiac remodeling upon pressure overload.

### Afterload-induced expression of fetal genes is independent of Malat-1

**Fig 3:**
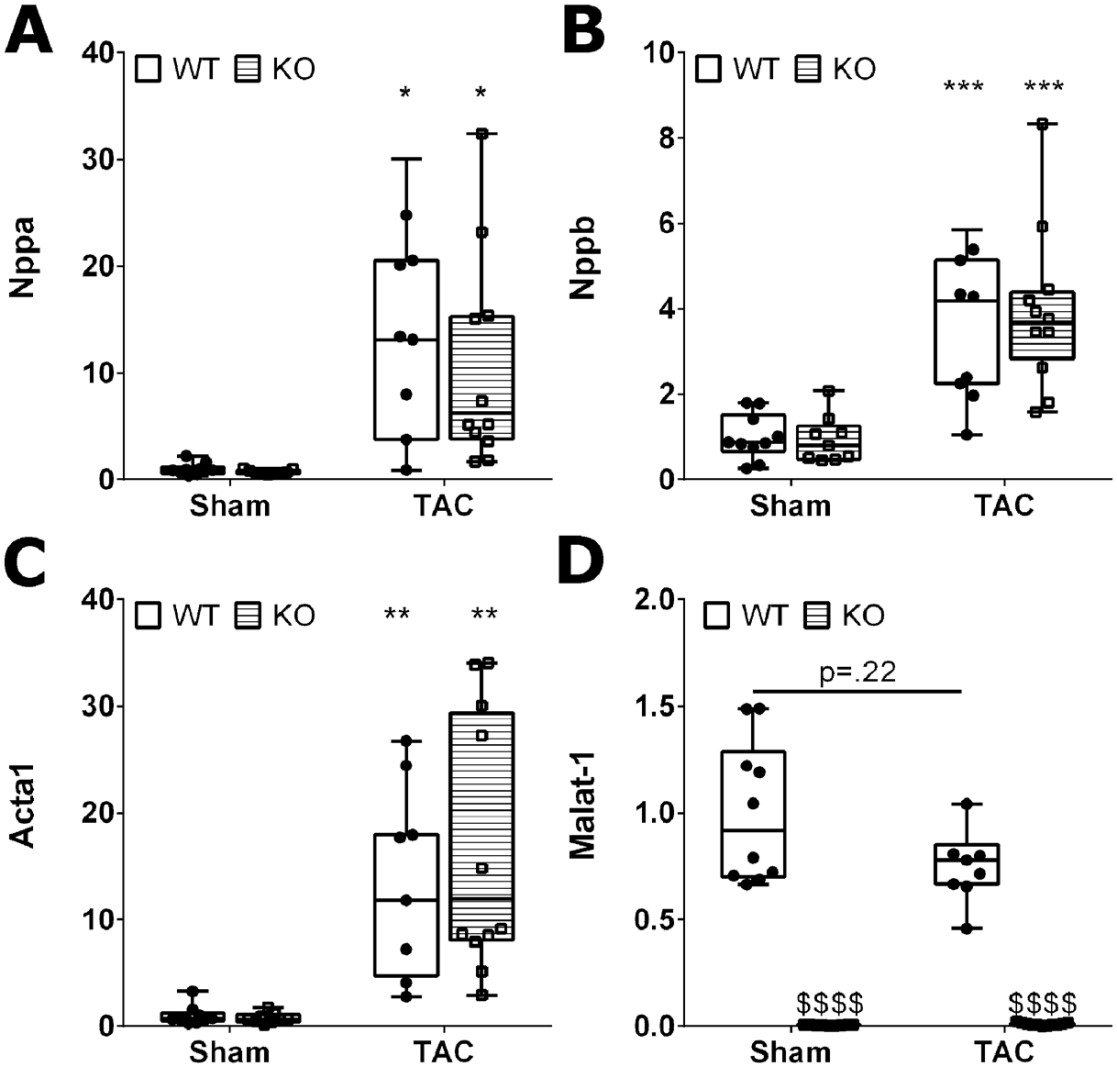
mRNA levels of hypertrophy markers. The hypertrophy marker genes *Nppa* (A), *Nppb* (B) and *Acta1* (C) were upregulated by TAC and not different between Malat-1 WT and KO mice. (D) Malat-1 itself was not significantly deregulated after TAC. *p<0.05, **p<0.01, ***p<0.001 TAC versus Sham;^$ $ $ $^p<0.0001 KO versus WT.

Cardiac hypertrophy goes along with the upregulation of mRNA levels of the natriuretic peptides A (*Nppa*) and B (*Nppb*) and the cytoskeletal protein skeletal alpha actin (*Acta1*) in the myocardium. We found mRNA levels of all three hypertrophy markers to be significantly increased after TAC, whereas ablation of Malat-1 did not interfere with upregulation of these genes (Fig 3A–3C).

**Fig 4:**
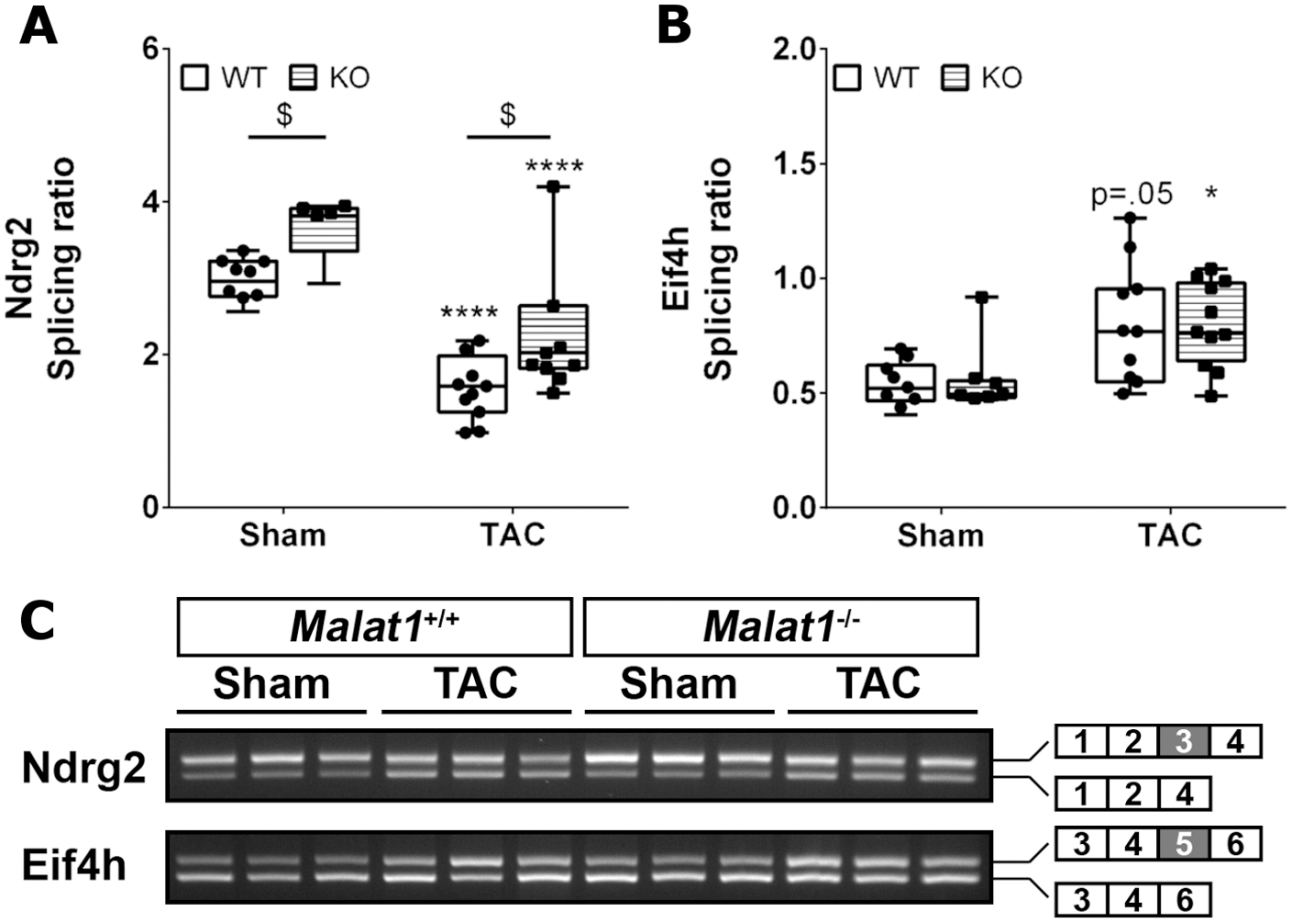
Alternative splicing of *Ndrg2* and *Eif4h* is evident after TAC. (A) Skipping of *Ndrg2* exon 3 is apparent after TAC in Malat-1 WT and KO mice, but absence of Malat-1 reduces this event both at baseline and after pressure overload. (B) Exon 5 inclusion of *Eif4h* is induced by TAC but not affected by absence of Malat-1. (C) Representative images of PCR products of *Eif4h* and *Ndrg2*. *p<0.05, ****p<0.0001 TAC versus Sham; ^$^p<0.05 KO versus WT.

Next to transcriptional changes, cardiac hypertrophy and failure also induce alternative splicing of certain mRNAs. We measured the fraction of alternatively spliced mRNA of *Ndrg2* and *Eif4h* and found that TAC as expected induced skipping of exon 3 of *Ndrg2*, as well as inclusion of exon 5 of *Eif4h* [31, 32]. Interestingly, *Ndrg2* showed a higher splice ratio in Malat-1 KO mice both with and without pressure overload (Fig 4A–4C) confirming a role of Malat-1 in splicing, whereas the splicing pattern of *Eif4h* was not different between genotypes.

## Discussion

Long non-coding RNAs are emerging as important players in several pathologic conditions, such as cancer and cardiovascular disorders. The nuclear lncRNA Malat-1 is exceptionally well-conserved among vertebrates and abundantly expressed in most organs and cell types investigated so far, implying an important role throughout evolution. However, different approaches to knockout Malat-1 in mice have shown no effect on development and normal life [15–17], leading researchers to assume that Malat-1 becomes relevant only under stressed conditions. Heart failure is the final end stage of many cardiac diseases and a major cause of death worldwide. We investigated the effect of genetic ablation of Malat-1 on molecular, histological, morphological, and functional changes during development of heart failure in two different mouse models. Although we confirm a role for Malat-1 in mRNA splicing, we found that this lncRNA has no crucial role in pressure overload induced cardiac hypertrophy and failure.

*In vivo*, Malat-1 abrogation was recently found to cause aberrant vessel growth in a model of hind limb ischemia [22]. However, the functional relevance of this finding was not assessed in the study. In the pressure-overloaded heart, a transient increase in capillary density and concomitant improved perfusion is indispensable for development of compensated hypertrophy and maintained cardiac function [19]. Therefore, we expected Malat-1 to accelerate heart failure development by impeding the compensatory response of capillary growth upon pressure overload. However, while four weeks of TAC resulted in heart failure with decreased capillary density, we found no role for Malat-1 in regulating cardiac capillary density both under normal physiological and under pressure overloaded conditions. Next to vessel growth, Malat-1 is essential for proliferation and migration of several cancer cell lines and was reported to activate the ERK/MAPK growth signaling pathway [29], which is well-known for its central role in cardiomyocyte hypertrophy. Additionally, Malat-1 has been proposed to act as a competing endogenous RNA for microRNA-133, thereby attenuating miR-133 mediated repression of serum response factor (SRF) [24]. The muscle-specific miR-133 has critical functions in the heart and is a powerful inhibitor of cardiac hypertrophy [25, 26, 33] and the transcription factor SRF is an important regulator of several hypertrophy associated genes, such as *Nppa*, *Nppb* and *Acta1* [34]. However, our findings indicate no role of Malat-1 on transcriptional changes or on overall cardiac hypertrophy during pressure overload. These findings argue against a relevant influence of Malat-1 on ERK/MAPK signaling or miR-133/SRF regulation in the heart.

Cardiac pro-hypertrophic signaling together with cardiomyocyte damage leads to the activation of resident fibroblasts and deposition of interstitial connective tissue. Indeed, both AngII and TAC induced myocardial collagen deposition and ablation of Malat-1 did not affect this process, ruling out an important role of this lncRNA in fibroblast activation. Additionally, the number of CD45^+^ cells in the pressure-overloaded heart was not affected by presence or absence of Malat-1, although a role for Malat-1 in pro-inflammatory cytokine production by HUVECS has recently been suggested [35]. On the cellular level, knockdown of Malat-1 results in alternative splicing of several genes [14], and in line with this, it was found that Malat-1 co-localizes with the splicing factors ASF/SF2 in nuclear speckles of mouse embryonic fibroblasts and cultured neurons [36]. Interestingly, a deficiency of ASF/SF2 has been shown to alter cardiomyocyte function by affecting splicing of calcium/calmodulin-dependent kinase IIδ [37] and perturbation of mRNA splicing is a feature of heart failure [38]. Interestingly, we found alternative splicing of *Ndrg2*, which shows skipping of exon 3 in hypertrophic mouse hearts [31], to be less common in Malat-1 KO mice both at baseline and after pressure overload. In contrast, alternative splicing of *Eif4h* was apparent after pressure overload as previously reported [32] but not affected by ablation of Malat-1. These data confirm that Malat-1 can influence splicing of individual mRNAs but do not indicate an important role of this effect in cardiac pressure overload. The named molecular and histological changes entail effects on cardiac morphology and function both in Malat-1 WT and KO mice. Our echocardiographic analysis is limited to systolic function, but in view of the comparable cardiomyocyte hypertrophy and myocardial fibrosis it appears unlikely that diastolic function is affected by absence of Malat-1. In conclusion, despite extensive phenotyping of cardiac function, morphology, histological appearance, and gene expression, no important differences could be found in the hearts of Malat-1 WT and KO mice after TAC- or AngII-induced cardiac pressure overload.

While this manuscript was in preparation, another group reported that inhibition of Malat-1 may have protective effects on LV dilation and dysfunction in a model of streptozotocin-induced diabetic cardiomyopathy. This was associated with dampening of cytokine expression and cardiomyocyte apoptosis [39, 40]. However, no information was given on possible cell types and signaling pathways responsible for this effect, which impedes a proper comparison with other findings. Above that, the power and relevance of these two short reports are difficult to assess due to the lack of information on data presentation and statistical analysis. Therefore, more detailed knowledge is needed to reconcile a possible role of Malat-1 in diabetic cardiomyopathy with its insignificance during pressure overload-induced cardiac hypertrophy and failure.

Interestingly, only an effect of Malat-1 on vascularization of the retina was shown in Malat-1 KO mice [22], whereas its functions in hind limb ischemia, ERK/MAPK signaling, miR-133 scavenging, and possibly diabetic cardiomyopathy were exclusively shown by posttranscriptional knockdown of Malat-1 [22–24, 29, 39, 40]. It is therefore conceivable that compensatory pathways are activated in Malat-1 KO mice during embryonic development that allow for normal cardiac function and adaptation in our study. Inducible knock-out strategies or deep RNA sequencing may help to circumvent and to identify possible compensatory mechanisms, respectively. Importantly, the nature of such compensatory changes would directly help to deduce the regular function of Malat-1. Above that, phenotypical changes upon deletion of a lncRNA can depend on the knockout strategy employed, which constitutes a major difficulty for investigating lncRNA functions *in vivo* [41]. Subtle phenotypical differences have been observed between the three different Malat-1 knockout lines generated so far [42] and it is therefore conceivable that the promoter or other parts of the Malat-1 locus may have a function independent of the actual Malat-1 transcript. However, our results clearly show that the Malat-1 lncRNA transcript is dispensable during pressure overload induced cardiac hypertrophy and dysfunction. While we cannot exclude transcript-independent functions of the Malat-1 locus or the existence of compensatory mechanisms, our findings suggest no important role for Malat-1 in heart failure.

## Conclusions

We deployed two mouse models of pressure overload-induced heart failure to investigate the function of the lncRNA Malat-1 in a highly relevant human disease. Despite its reported function as regulator of vascularization, activator of ERK/MAPK signaling, and scavenger for the muscle-specific miR-133, we conclude that Malat-1 has no important role for cardiac hypertrophy and failure *in vivo*. Our findings therefore stress the importance of validating proposed lncRNA functions in clinically relevant disease models.

## Supporting Information

S1 Fig Gross morphological and functional analysis of Malat-1 WT and KO hearts after AngII infusion. Increased heart weight/body weight (A) and diastolic left ventricular wall thickness (B) without effects on diastolic left ventricular inner diameter (C) indicate concentric hypertrophy in both Malat-1 WT and KO mice. Decreased fractional shortening (D) without increased lung weight/body weight (E) indicate beginning of heart failure independent of Malat-1 deficiency. (F) Representative photographs of mouse hearts, Scale bar: 1 cm; *p<0.05, **p<0.01, ***p<0.001 AngII versus Sham.
(DOCX)

S2 Fig Histological analysis of LV and septal myocardium after AngII. No significant differences between WT and Malat-1 KO mice were found regarding cardiomyocyte hypertrophy (A), interstitial fibrosis (B), endocardial capillary density (C), or leucocyte infiltration (D). *p<0.05, **p<0.01 AngII versus Sham. Scale bars: Sirius Red: 1 mm; all other stains: 100 μm.
(DOCX)

S1 Table Characteristics of Malat-1 WT and KO mice after sham surgery or pressure overload. (DOCX)

S2 Table Complete dataset used to generate the graphs. (XLSX)
