# Supplementary material for: Long Non-Coding RNA Malat-1 Is Dispensable during Pressure Overload-Induced Cardiac Remodeling and Failure in Mice
Source: PLoS One. 2016 Feb 26;11(2):e0150236. doi: 10.1371/journal.pone.0150236 (PMC4769011; doi:10.1371/journal.pone.0150236)
Supplement: S1 Fig — Increased heart weight/body weight (A) and diastolic left ventricular wall thickness (B) without effects on diastolic left ventricular inner diameter (C) indicate concentric hypertrophy in both Malat-1 WT and KO mice. Decreased fractional shortening (D) without increased lung weight/body weight (E) indicate beginning of heart failure independent of Malat-1 deficiency. (F) Representative photographs of mouse hearts, Scale bar: 1 cm; *p<0.05, **p<0.01, ***p<0.001 AngII versus Sham. (DOCX) [file pone.0150236.s001.docx]

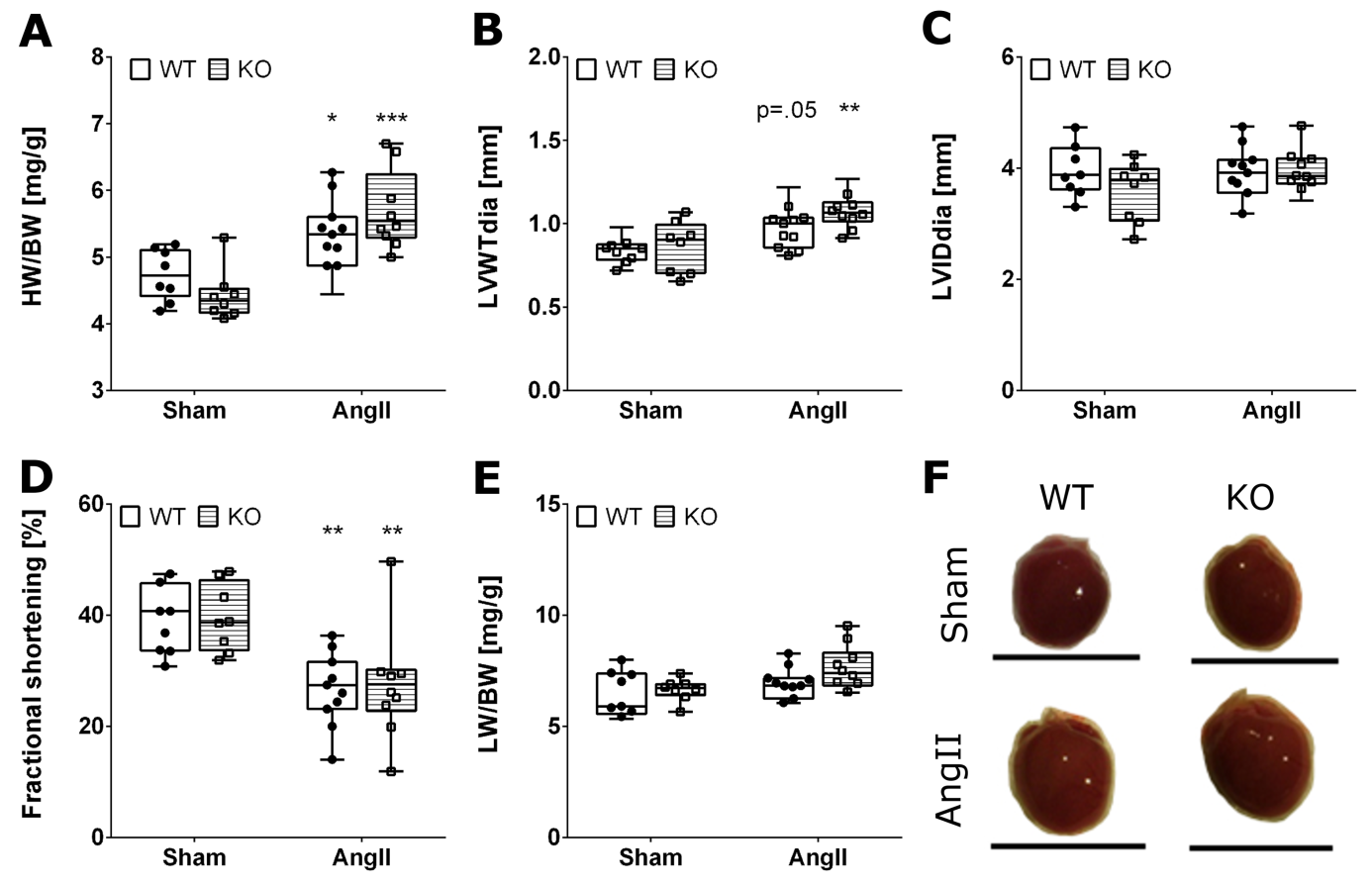


S1 Fig: Gross morphological and functional analysis of Malat-1 WT and KO hearts after AngII infusion. Increased heart weight/body weight (A) and diastolic left ventricular wall thickness (B) without effects on diastolic left ventricular inner diameter (C) indicate concentric hypertrophy in both Malat-1 WT and KO mice. Decreased fractional shortening (D) without increased lung weight/body weight (E) indicate beginning of heart failure independent of Malat-1 deficiency. (F) Representative photographs of mouse hearts, Scale bar: 1 cm; *p<0.05, **p<0.01, ***p<0.001 AngII versus Sham.
