# Supplementary material for: Long Non-Coding RNA Malat-1 Is Dispensable during Pressure Overload-Induced Cardiac Remodeling and Failure in Mice
Source: PLoS One. 2016 Feb 26;11(2):e0150236. doi: 10.1371/journal.pone.0150236 (PMC4769011; doi:10.1371/journal.pone.0150236)
Supplement: S2 Fig — No significant differences between WT and Malat-1 KO mice were found regarding cardiomyocyte hypertrophy (A), interstitial fibrosis (B), endocardial capillary density (C), or leucocyte infiltration (D). *p<0.05, **p<0.01 AngII versus Sham. Scale bars: Sirius Red: 1 mm; all other stains: 100 μm. (DOCX) [file pone.0150236.s002.docx]

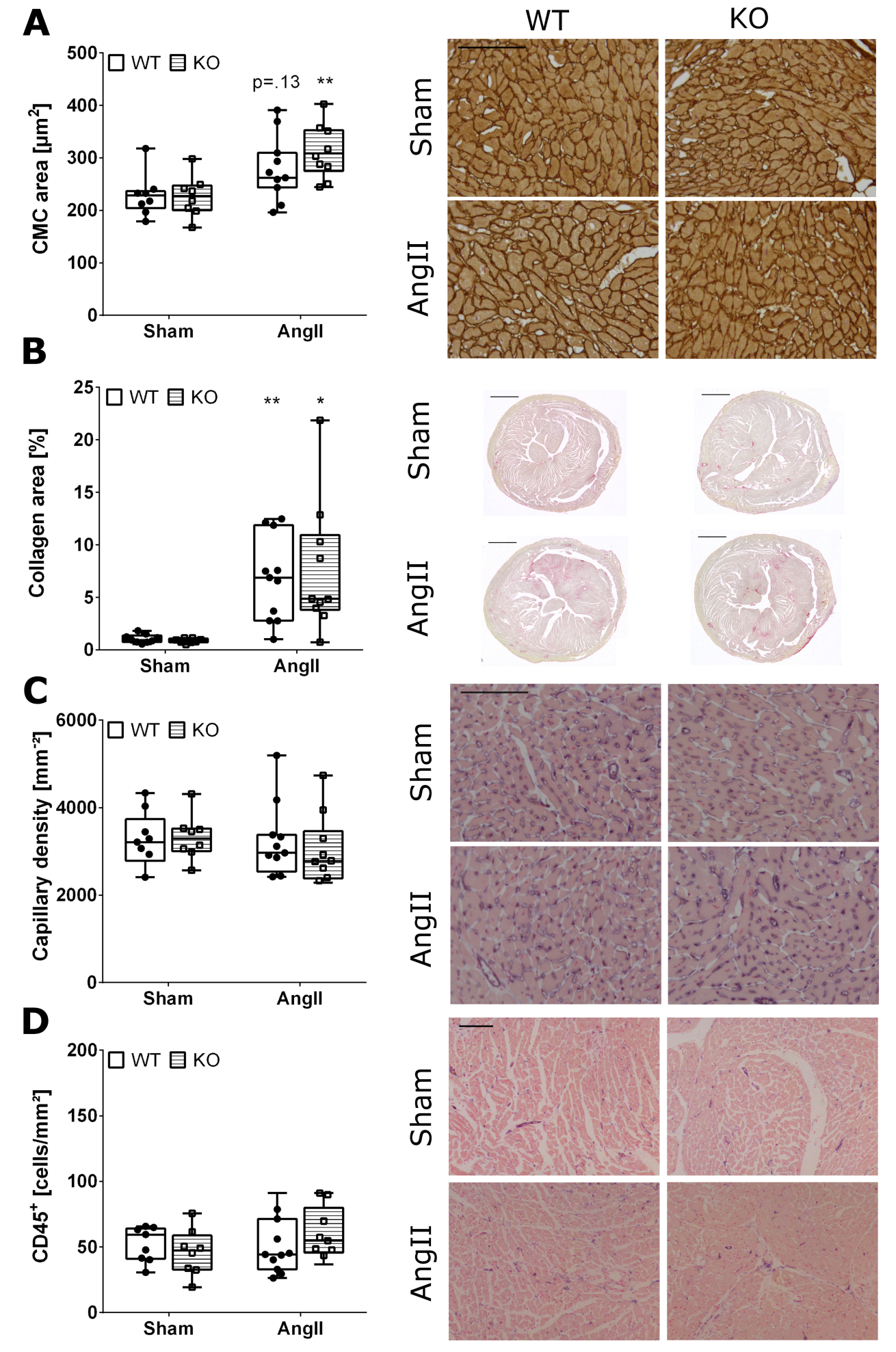


S2 Fig: Histological analysis of LV and septal myocardium after AngII. No significant differences between WT and Malat-1 KO mice were found regarding cardiomyocyte hypertrophy (A), interstitial fibrosis (B), endocardial capillary density (C) or leucocyte infiltration (D). *p<0.05, **p<0.01 AngII versus Sham. Scale bars: Sirius Red: 1 mm; all other stains: 100 µm.
