# Supplementary material for: Long Non-Coding RNA Malat-1 Is Dispensable during Pressure Overload-Induced Cardiac Remodeling and Failure in Mice
Source: PLoS One. 2016 Feb 26;11(2):e0150236. doi: 10.1371/journal.pone.0150236 (PMC4769011; doi:10.1371/journal.pone.0150236)
Supplement: S1 Table — (DOCX) [file pone.0150236.s003.docx]

S1 Table: Characteristics of Malat-1 WT and KO mice after sham surgery or pressure overload.
